# Women’s Midlife Health: Why the Midlife Matters

**DOI:** 10.1186/s40695-015-0006-7

**Published:** 2015-08-11

**Authors:** Siobán D. Harlow, Carol A. Derby

**Affiliations:** 1 Department of Epidemiology, School of Public Health, University of Michigan, Ann Arbor, Michigan USA; 2 Department of Epidemiology, Albert Einstein College of Medicine, Bronx, New York USA

**Keywords:** Women, Midlife, Menopause, Menopausal transition, Health

## Abstract

ᅟ

With the launch of *Women’s Midlife Health,* we seek to stimulate increased scientific and clinical focus on the midlife and its relevance to health and healthy aging. Over the past two decades, several cohort studies have advanced scientific understanding of the natural history of ovarian aging and begun to elucidate the inter-relationship between ovarian aging and, for example, bone, cardiovascular, cognitive, and musculoskeletal health. Notably, these studies have also shed light on age-related changes that occur in midlife independent of ovarian aging. Critical insights include observations that bone loss, osteoarthritis, adverse changes in lipid profiles, diabetes, metabolic syndrome, and sleep disturbances frequently begin and/or accelerate during the midlife period – in some cases accelerated by the endocrine changes associated with the menopause and in others simply coincident with them. It is apparent that healthy behaviors in midlife, such as maintenance of physical activity and healthy body weight may moderate these changes. Furthermore, it is increasingly recognized that healthy lifestyles and control of vascular risk factors in midlife may be beneficial for cognitive health in later years. Thus, the evidence strongly suggests that the midlife represents a critical window for preventing chronic disease and optimizing health and functioning.

In addition to symptoms that are the hall mark of menopause, i.e. vasomotor symptoms or “hotflashes”, several health conditions peak or are exacerbated during the midlife, including fibroids and consequent hysterectomies, migraine and systemic lupus erythematosus. Thus the midlife also warrants attention as a vulnerable window for an important subset of gynecologic and hormone-sensitive conditions.

We define the midlife as beginning at about age 35–40 years and extending to about age 60–65 years. This age range encompasses the late reproductive to late postmenopausal stages of reproductive aging [1–4]. Thus, for women, it includes the menopausal transition and post-menopause up through the age where, presumably, ovarian senescence is no longer actively influencing the aging process. Studies of aging have typically focused on and enrolled participants aged 60 or 65 and older, an age when the window of opportunity for primary prevention may well have been missed. While prevention and healthy lifestyles are important at all ages, it is increasingly apparent that health in midlife is an important determinant of a healthy and fully functional life in the following decades.

For example, the mid-life appears to be a vulnerable window for onset of functional limitations and the transition to disability. Up to one quarter of recently menopausal women report having substantial functional limitations. Mobility-based disabilities are highly prevalent, with one midlife cohort reporting that 39 % of women reported moderate to extreme difficulty in mobility as measured by the World Health Organization Disability Assessment Schedule [5]. Declines in functioning during this life-stage may be related to the acceleration of other adverse changes in the midlife including the onset of metabolic syndrome and diabetes, adverse changes in lipid profiles, hearing and vision, and body composition changes but may also be a direct result of the onset of osteoarthritis. In one of few prospective studies that included midlife women, two-thirds had evidence of osteoarthritis, the leading cause of pain and adult disability in the United States, in the early post-menopause (mean age 57 years) [6].

Risk of osteoporosis and, concomitantly, fracture increases with aging and it has long been understood that bone loss is associated with menopause. The midlife cohort studies have now documented that bone loss begins prior to the final menstrual period with an accelerated period of loss in bone mineral density and bone strength beginning 1–2 years before menopause until 2–5 years into the post-menopause[7]. As it is estimated that about half of the lifetime loss in bone mineral density occurs during this relatively short period in the midlife, it represents a critical period for interventions to optimize reductions in the loss of bone strength.

The Women’s Health Initiative refocused attention on the midlife as potentially a vulnerable window for interventions to protect heart health with a call for increased research on change in women’s cardiovascular risk profile during the menopausal transition. Recent studies support the hypothesis that increase in cardiovascular risk accelerates with ovarian aging. The Study of Women’s Health Across the Nation has documented that levels of total cholesterol, low density lipoprotein and apolipoprotein B, increase significantly in the 2-year window around the menopause [8] as do adverse changes in measures of aortic calcification during the late menopausal transition [9]. Notably, emerging evidence suggests that the hallmark menopausal symptom, the hotflash, is associated with increased cardiovascular risk profiles [10] with several ongoing studies working to identify the mechanisms underlying these associations. Ongoing studies will provide data regarding the trajectories of these changes in cardiovascular risk in late post-menopause.

Understanding depression risk across the life-course also requires increased insight into the changes in risk that occur at midlife. Approximately 20-30 % of mid-life women experience incident or recurrent episodes of clinical depression, the leading cause of health-related disability in women. Fluctuations in reproductive hormones associated with ovarian aging are one potential mechanism underlying midlife risk, however, other menopause-associated physiological and psychosocial changes including increased prevalence of sleep disturbances likely also play a role.

In sum the relative lack of attention to midlife health ignores the evidence that critical changes are occurring during this life-stage that warrant changes in lifestyle, behavior, social engagement and health care practices. As suggested by the old saying -- *At 40, your eyesight starts to go; at 50 everything else starts to go –* the midlife is a period of substantial physiologic change that requires adaptive change to optimize health and functioning. The goal of *Women’s Midlife Health* is to provide a forum for increased scientific inquiry into the mechanisms that underlie the physiologic changes of the midlife, the triggers for and nature of the aging-and ovarian-aging related processes that are initiated during this life-stage, and the critical factors, particularly modifiable ones, that influence the risk of healthy versus unhealthy aging. Given the increasing wealth of knowledge about the impact of ovarian senescence, research on non-ovarian triggers of symptomatic and non-symptomatic aging-related physiologic changes is a specific priority. Comparative studies of the midlife in men are also warranted.

Notably, data on the midlife are particularly scant from low and middle income countries, where optimizing health during this life-stage will be critical to enhancing life expectancy and reducing disability. Evaluation of context specific health risks more common in such settings, such as the impact of late childbirth and life-long under-nutrition, is warranted. Also lacking are data regarding racial/ethnic differences in midlife health, and whether trajectories of change during the menopause transition differ.

*Women’s Midlife Health* welcomes clinical and population-based research that provides new insights into the health transitions that occur during midlife and with menopause, with a particular interest in articles addressing vulnerabilities and opportunities during this life stage and addressing interventions that promote a healthy transition and healthy aging. Manuscripts that consider the impact of the environment and occupation, areas frequently ignored in studies of women, as well as gender-specific social factors on health and disease processes during this life-stage are encouraged.
